# Omitted Conflict of Interest Disclosures

**DOI:** 10.1001/jamahealthforum.2024.3106

**Published:** 2024-08-09

In the Viewpoint titled “Reforming 340B to Serve the Interests of Patients, Not Institutions,”^1^ published online July 26, 2024, an author’s disclosed conflicts of interest were inadvertently omitted during the editing process. The Conflict of Interest Disclosures section has been updated to include, “Dr Winegarden’s employer receives funding for policy research from the pharmaceutical industry, but he did not receive any direct funding for this Viewpoint.” This article was corrected online.
